# A rangewide herbarium‐derived dataset indicates high levels of gene flow in black cherry (*Prunus serotina*)

**DOI:** 10.1002/ece3.4719

**Published:** 2019-01-08

**Authors:** Lauren Konrade, Joey Shaw, James Beck

**Affiliations:** ^1^ Department of Biology Wichita State University Wichita Kansas; ^2^ Department of Biology, Geology, and Environmental Science University of Tennessee Chattanooga, Chattanooga Tennessee; ^3^ Botanical Research Institute of Texas Fort Worth Texas

**Keywords:** herbarium DNA, isolation by distance, microsatellites, North America, *Prunus serotina*, Rosaceae

## Abstract

Isolation by Distance (IBD) is a genetic pattern in which populations geographically closer to one another are more genetically similar to each other than populations which are farther apart. Black cherry (*Prunus serotina* Ehrh.) (Rosaceae) is a forest tree species widespread in eastern North America, and found sporadically in the southwestern United States, Mexico, and Guatemala. IBD has been studied in relatively few North American plant taxa, and no study has rigorously sampled across the range of such a widespread species. In this study, IBD and overall genetic structure were assessed in eastern black cherry (*P. serotina* Ehrh. var. *serotina*), the widespread variety of eastern North America. Eastern North America. *Prunus serotina* Ehrh. var. *serotina* (Rosaceae). Dense sampling across the entire range of eastern black cherry was made possible by genotyping 15 microsatellite loci in 439 herbarium samples from all portions of the range. Mantel tests and STRUCTURE analyses were performed to evaluate the hypothesis of IBD and genetic structure. Mantel tests demonstrated significant but weak IBD, while STRUCTURE analyses revealed no clear geographic pattern of genetic groups. The modest geographic/genetic structure across the eastern black cherry range suggests widespread gene flow in this taxon. This is consistent with *P. serotina*'s status as a disturbance‐associated species. Further studies should similarly evaluate IBD in species characteristic of low‐disturbance forests.

## 1 INTRODUCTION

Gene flow, the movement of genes among populations, is a fundamental aspect of evolution within plant populations, either alone or in concert with genetic drift, mutation, and selection (Ellstrand, 2014). Particularly in widespread species, the geographic distance between populations leads to an intuitive expectation of decreasing gene flow between populations as the distance between these populations increases. Isolation by Distance (IBD) is the corresponding biogeographic expectation, in which a given population is most genetically similar to geographically proximate populations, with decreasing similarity to increasingly distant populations (Sexton, Hangartner, & Hoffmann, 2013; Wright, 1943). Although evaluating this pattern is analytically straightforward, IBD has been rigorously assessed in relatively few widespread North American plant taxa, among the forest trees. The main obstacle to geographically thorough tests of IBD is the difficulty in assembling a sample set representative of an entire species' range. While a few studies have analyzed large sets of samples, these are often drawn from a relatively small number (<100) of locations (Campitelli & Stinchcombe, 2013; Griffin & Barrett, 2004; Hoban et al., 2010; Mylecraine, Kuser, Smouse, & Zimmermann, 2004; Parker, Hamrick, Parker, & Stacy, 1997; Waselkov & Olsen, 2014) and/or from a subset of the total species range (Dennhardt, DeKeyser, Tennefos, & Travers, 2016; Hadziabdic, 2010; Lloyd, Roche, & Roberts, 2011; Yakimowski & Eckert, 2008). This lack of sampling is due to feasibility, as field collecting material across large spatial scales is an expensive and extremely time‐consuming task (planning routes, obtaining permits, fieldwork, preparing voucher specimens, permit reporting). Field collecting is therefore often done in a highly non‐random manner, with sampling confined to a few groups of geographically clustered populations. As a result, our current understanding of the geographic structure of genetic variation in widespread North American plants is based on uneven sampling designs.

Previous studies do suggest that IBD is present, at least in native North American plant species. In herbaceous plants, Waselkov and Olsen (2014) demonstrated a significant (*R* ^2^ = 0.079) relationship in waterhemp [*Amaranthus tuberculatus* (Moq. ex DC) J. D. Sauer] with a 10‐locus microsatellite dataset, Griffin and Barrett (2004) found significant IBD in *Trillium grandiflorum* (Michx.) Salisb. (*R* ^2^ *= *0.17) using allozymes, and a study of 10 microsatellite loci found a significant signal of IBD in diploid *Galax urceolata* (Poir.) Brummitt (*R* ^2^ = 0.148) (Servick, Visger, Gitzendanner, Soltis, & Soltis, 2015). Significant IBD has also been observed in widespread North American woody plants. Hoban et al. (2010) reported significant IBD in butternut (*Juglans cinera* L.) with 11 microsatellite loci (*R* ^2^ = 0.128/*R* ^2^ = 0.067 *F*_ST_/*R*_ST_), as did Geraldes et al. (2014) with SNPs in black cottonwood (*Populus trichocarpa* Torr. & A. Gray ex Hook) (*R* ^2^ = 0.2873). Victory, Gloubitz, Rhodes, and Woeste (2006) also found a significant, albeit weak, signal of IBD in *Juglans nigra* using 12 microsatellite loci (*R* ^2^ = 0.006), as did Hadziabdic (2010) in flowering dogwood (*Cornus florida*) (*R* ^2^ = 0.01). Many other studies have indirectly addressed the notion of IBD in North American plants, often through the visual interpretation of the geographic array of DNA sequence haplotypes or multivariate genetic clusters. Thirteen of the 19 native plant taxa reviewed in Soltis, Morris, McLachlan, Manos, and Soltis (2006) exhibited some level of geographic structure, as have numerous subsequent studies (Morris, Ickert‐Bond, Brunson, Soltis, & Soltis, 2008; Rodrigues & Stefanovic, 2016; Thomson, Dick, & Dayanandan, 2015; Tsai & Manos, 2010; Willyard et al., 2017; Zinck & Rajora, 2017). In contrast, many exotic species in North America do not exhibit IBD. Jorgensen and Maruicio (2004) found no signal in North American *Arabidopsis thaliana* (L.) Heynh. (*p* = 0.99) with amplified fragment length polymorphisms (AFLPs), and Kirk, Paul, Straka, and Freeland (2011) and Stabile et al. (2016) found no signal of IBD in North American *Phragmites australis* (Cav.) Trin. ex Steud. (*p* = 0.979 and 0.62, respectively) using microsatellite markers. Durka, Bossdorf, Prati, and Auge (2005) did find a significant but weak signal of IBD in *Alliaria petiolata* Cavara & Grande (*p* = 0.002, *R* ^2^ = 0.043) with microsatellite loci. This general lack of IBD in exotic species is not surprising given the potential for multiple introductions of genetically variable material to the non‐native range, and the propensity for widespread non‐native species to exhibit frequent long‐range gene flow.

Previous research therefore suggests that IBD is to be expected in native North American plants, and this study aims to test this basic hypothesis of genetic structure in eastern black cherry (*Prunus serotina* Ehrh. var. *serotina*), a widespread eastern North American forest tree that is important ecologically as a wildlife food source (Thompson & Willson, 1979) and as timber (Auclair & Cottam, 1971). Each year over 7 million board feet of black cherry are harvested (Howard & Westby, 2013), largely for use as a veneer in furniture making (Gatchell, 1971). Eastern black cherry has by far the largest range of the five *P. serotina* varieties, found throughout the United States and southern Canada east of 96° west longitude (McVaugh, 1951), with rare occurrences in Mexico and Guatemala. The remaining varieties have more restricted distributions*. Prunus serotina* Ehrh. var. *eximia* (Small) Little is restricted to the Edwards Plateau of central Texas; *Prunus serotina* Ehrh. var. *alabamensis* (C. Mohr) Little is found in Georgia, Alabama, South Carolina, and Florida; *Prunus serotina* Ehrh. var. *salicifolia* (Kunth) Koehne is found in southern Mexico and Guatemala; and *Prunus serotina* Ehrh. var. *rufula* (Wooton & Standl.) McVaugh from Texas to Guanajuato (McVaugh, 1951).

As described above, obtaining a geographically representative set of eastern black cherry samples would not be feasible through traditional fieldwork. In this study, we bypass this time limitation through the extensive use of museum tissue. Black cherry is commonly collected, and thousands of specimens are archived in North American herbaria. Specimen locality data allow for the rapid choice of a geographically representative set of specimens from which a genetic dataset can be obtained.

## 2 MATERIALS AND METHODS

### 2.1 Obtaining samples

Our group personally visited 21 herbaria (obtaining 373 specimens) and requested tissue from 13 herbaria (131 specimens) in 2014, 2015, and 2016 (see Acknowledgments and Table S1 in Appendix S1). Following visits to large national institutions, specific regional and local collections were targeted in order to address geographic gaps in our sample set. We attempted to choose one specimen per county, generally avoiding adjacent counties. All identifications were verified, with a positive identification for *P. serotina* var. *serotina* involving two or more of the following characteristics when possible: small orange hairs at the base of the midrib, (hooked) serrated leaf margins, leaves more than 2.5× as long as wide, and persistent sepals when in fruit. One or two small leaves were removed when sufficient material was present, and labels noting that material was removed for DNA extraction were affixed to all sampled sheets. All specimens were georeferenced with Google Earth (Google Inc, 2009) and EarthPoint (Clark, 2015) software. All specimen locality data can be found in Dryad (https://doi.org/10.5061/dryad.r1f08nt).

### 2.2 Molecular methods

Following a high‐throughput DNA extraction (Beck et al., 2012), 15 microsatellite loci originally designed for other *Prunus* species were amplified (Table 1): (Cipriani et al., 1999; Dirlewanger et al., 2002; Downey & Iezzoni, 2000; Germain‐Aubrey, Soltis, Soltis, & Gitzendanner, 2011; Struss, Ahmad, Southwick, & Boritzki, 2003; Testolin et al., 2000; Wang, Walla, Zhong, Huang, & Dai, 2012; Yamamoto et al., 2002). These loci were chosen on the basis of previous success in *Prunus serotina* var. *serotina* (Pairon & Jaquemart, 2008; Pairon et al., 2010) or preliminary CAG‐tag PCR screening (Beck et al., 2014). Three to four‐locus sets (Table 1) were dye‐labeled and simultaneously amplified in 8 μl reactions using the following reagents: 2.5 μl Qiagen multiplex PCR Master mix (Qiagen, Germantown, MD), 2 μM each primer, and 2 μl 1:10 diluted DNA template. PCR cycling conditions included an initial denaturing step at 95°C for 15 min, 30 cycles of 94°C for 30 s/53°C for 1 min and 30 s/72°C for 1 min, followed by a final extension at 60°C for 30 min. Amplicons were sized at the University of Chicago Comprehensive Cancer Center DNA Sequencing and Genotyping Facility, and alleles were scored with GeneMarker 1.9 (SoftGenetics, 2012). As a quality control measure, 20% of samples were both re‐extracted and re‐amplified, and an additional 14% were re‐amplified from existing extractions. All genotypes included in the study can be found in Dryad (https://doi.org/10.5061/dryad.r1f08nt).

**Table 1:** The 15 microsatellite loci used in this study. “Diploid” refers to a primer set's amplification of both genomes of the allotetraploid *P. serotina* var.* serotina* at that locus (No) or only one of these two genomes (Yes)

Locus name	Locus set	Publication	Original target species	Diploid	Dye	Number of alleles	Private alleles
UDP96‐005	1	Cipriani et al. (1999)	*Prunus persica*	Yes	6‐FAM	7	0
UCD‐CH14	1	Struss et al. (2003)	*Prunus avium*	Yes	6‐FAM	18	1
UDP98‐405	1	Cipriani et al. (1999)	*Prunus persica*	Yes	HEX	6	0
M4c	2	Yamamoto et al. (2002)	*Prunus persica*	No	6‐FAM	15	3
PceGA34	2	Downey and Iezzoni (2000)	*Prunus cerasus*	Yes	6‐FAM	24	7
M12a	2	Yamamoto et al. (2002)	*Prunus persica*	No	HEX	34	2
UDP98‐025	3	Testolin et al. (2000)	*Prunus persica*	Yes	6‐FAM	16	5
pchpgms3a	3	Downey and Iezzoni (2000)	*Prunus persica*	Yes	6‐FAM	16	4
pchpgms3b	3	Downey and Iezzoni (2000)	*Prunus persica*	Yes	6‐FAM	13	1
pchgms2	3	Downey and Iezzoni (2000)	*Prunus persica*	No	HEX	15	4
UDP96‐001	3	Testolin et al. (2000)	*Prunus persica*	No	NED	11	3
BPPCT‐002a	4	Dirlewanger et al. (2002)	*Prunus persica*	Yes	6‐FAM	5	1
BPPCT‐002b	4	Dirlewanger et al. (2002)	*Prunus persica*	Yes	6‐FAM	9	3
C3292	4	Wang et al. (2012)	*Prunus virginiana*	Yes	HEX	7	1
BPPCT‐017	4	Dirlewanger et al. (2002)	*Prunus persica*	No	NED	29	11

### 2.3 Genotyping success assessment

To determine if specimen age or curatorial conditions affected genotyping success, we explored the relationship between the number of loci genotyped with both age of the specimen and its herbarium of origin. A generalized linear model implemented in R (R Core Team, 2015) was used. A generalized linear model (“glm” function) was constructed with the “poisson” distribution argument due to the non‐normal distribution of our dataset. A likelihood ratio test was then used to evaluate the fit of nested statistical models (“drop1” function with the “Chisq” test argument) to discover any significant relationship between specimen age, herbarium, and number of loci genotyped.

### 2.4 Mantel tests and structure analyses

Isolation by Distance was evaluated using Mantel tests (Mantel, 1967), which assessed the overall correlation between matrices of genetic and geographic distances among samples. Mantel tests were performed in R v.3.2.3 (R Core Team, 2015) using the package “vegan” (Oksanen et al., 2016) on three datasets: including only samples from which ≥10 loci were amplified (*n* = 464), including only samples from which ≥12 loci were amplified (*n* = 439), and including only samples from which ≥14 loci were amplified (*n* = 381). Mantel tests on all datasets detected similar levels of IBD (see results), and the “≥12‐locus dataset” was chosen for the remainder of our analyses to strike a balance between missing data and sample number. Mantel tests on subsets of the ≥12‐locus dataset to rule out effects of age and quality were also conducted. These subsets included an “old” dataset (individuals collected >33 years ago), a “young” dataset (individuals collected <33 years ago), and a “quality” dataset (individuals collected from relatively ecologically intact locations in which disturbance was minimal). “Quality” locations were those with relatively specific location information which did not note obviously anthropogenically altered sites (roadsides, agricultural landscapes, etc.). A Mantel correlogram analysis (Oden & Sokal, 1986), which seeks to find a correlation between genetic and geographic distances between samples separated by given geographic distance classes, was also conducted. Distance classes of 200 km each (e.g., all samples separated by 0–200 km, all samples separated by 201–400 km, etc.) were specified. Although all samples are presumably allotetraploid (Pairon & Jacquemart, 2008; Pairon & Jaquemart, 2005; Stairs & Hauck, 1967), 10 of the 15 loci amplified ≤2 alleles per sample, presumably due to mutational differences at priming sites in the two constituent genomes. Both standard Mantel and Mantel correlogram analyses utilized the basic “Lynch” band‐sharing distance (Lynch, 1990) obtained in the R package “polysat” v.1.7 (Clark & Jasieniuk, 2011). Other potentially applicable distance metrics require the addition of virtual alleles or the assumption of a stepwise mutation model and have been shown to distort distance matrices under certain parameter choices (Dufresne, Stift, Vergilino, & Mable, 2014). Finally, the presence and location of major genetic groups were evaluated with STRUCTURE v.2.3.4 (Hubisz, Falush, Stephens, & Pritchard, 2009). STRUCTURE attempts to identify populations that are in linkage equilibrium, and in the process assigns each sample to one of these populations or partially to multiple populations. STRUCTURE analyses were conducted on the ≥12‐locus dataset in two ways: including only the 10 loci exhibiting ≤2 alleles per locus per individual and including all loci "diploidized" (subsampled to include only two alleles per locus per individual) in GenoDive (Merimans & Van Tienderen, 2004). Both analyses included five iterations each at *K* = 2 to *K* = 10, with each iteration featuring 100,000 burnin generations followed by 500,000 data collection generations. All STRUCTURE analyses assumed the admixture and independent allele frequency models. The approach outlined in Evanno, Regnaut, and Goudet (2005) and implemented in Structure Harvester (Earl & vonHoldt, 2012) was used to find the most likely value of *K*.

## 3 RESULTS

### 3.1 Genetic dataset and genotyping success

**Figure 1:**
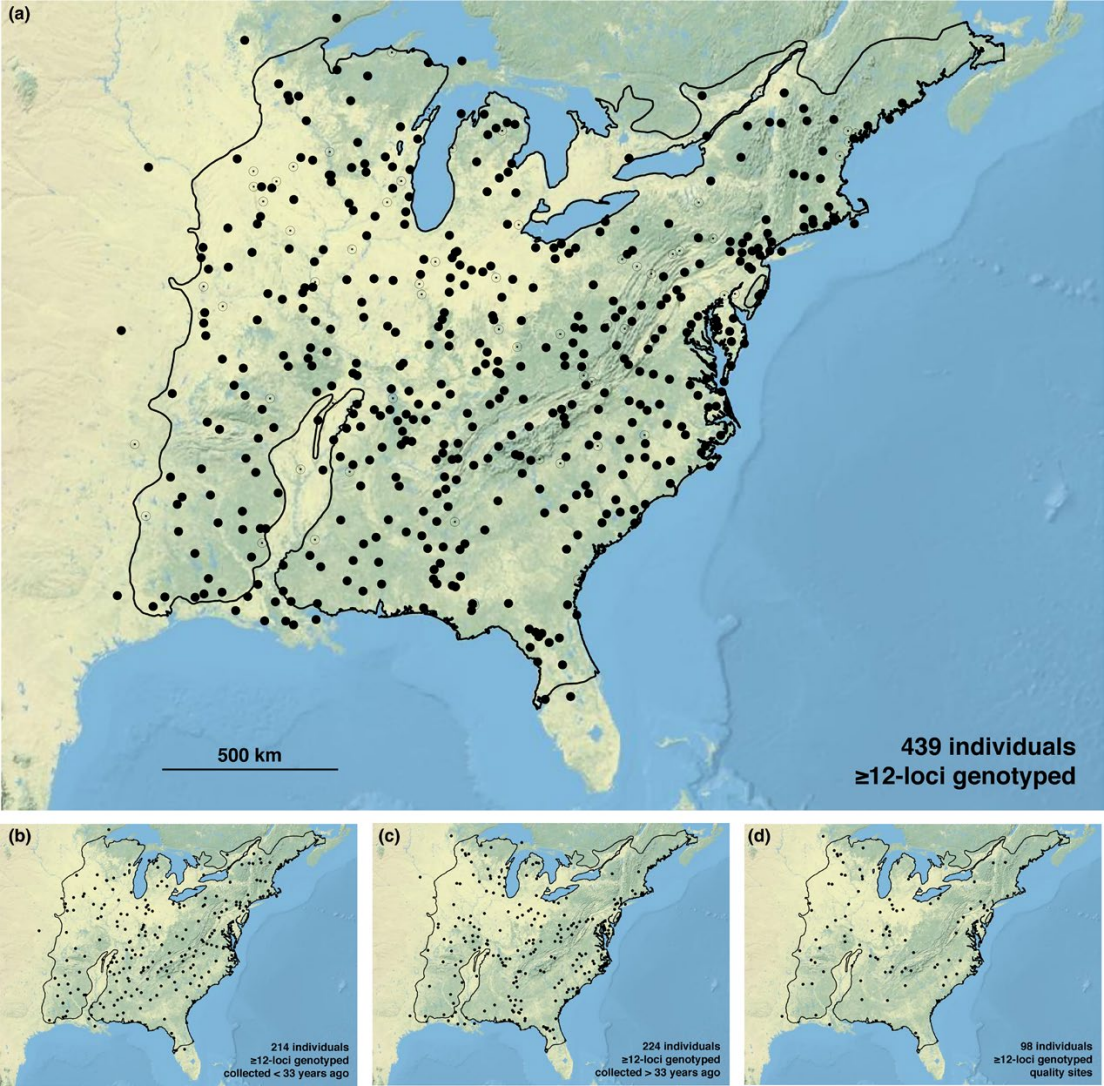
(a) Locations of 506 *Prunus serotina* var. *serotina* tissue samples obtained from 34 herbaria (Supporting Information Appendix S1). Dark circles indicate the 439 samples from which ≥12‐loci were amplified, open circles with a centered dot indicate 67 samples from which <12‐loci were amplified. (b) Location of 214 samples from which ≥12‐loci were amplified that were collected <33 years ago. (c) Location of 224 samples from which ≥12‐loci were amplified that were collected >33 years ago. (d) Location of 98 samples from which ≥12‐loci were amplified that were collected from relatively intact forest sites

**Figure 2:**
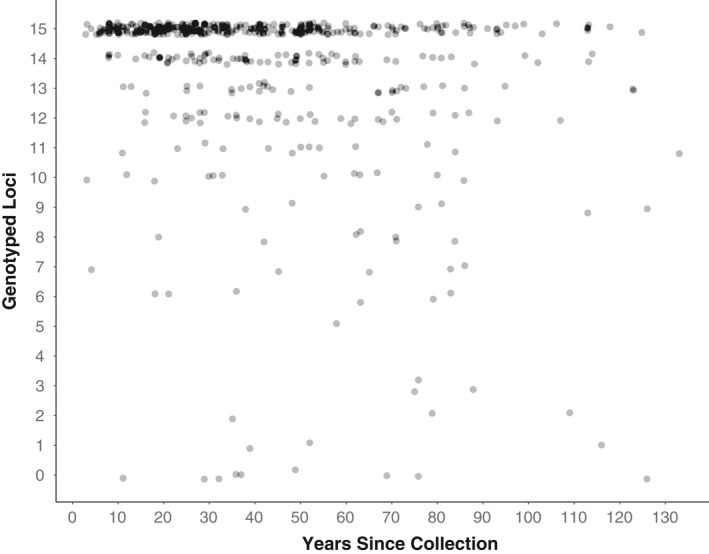
Microsatellite genotyping success versus specimen age in 506 *Prunus serotina* susbp. *serotina* samples. Plot of the number of loci genotyped (maximum 15) versus the years since the specimen was collected. Individual points have been slightly offset (the “jitter” function) to aid visibility

**Figure 3:**
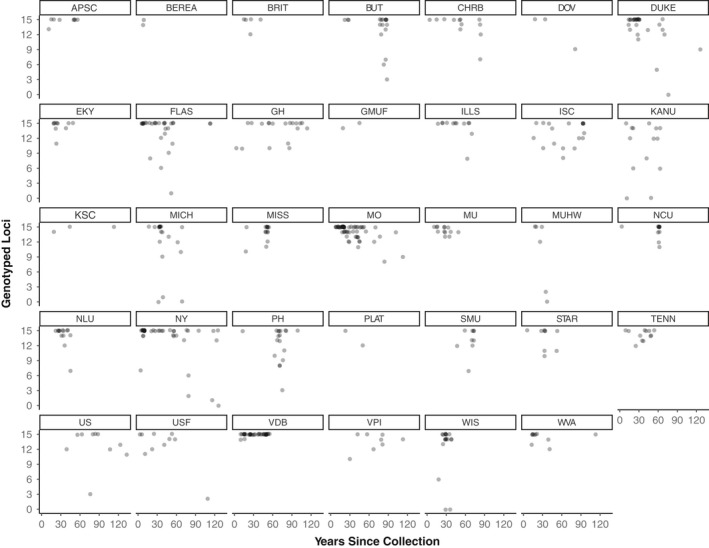
Microsatellite genotyping success versus specimen age in each of the 34 herbaria from which specimens were obtained. Individual points have been slightly offset (the “jitter” function) to aid visibility

The 506 *P. serotina* var. *serotina* samples obtained from 34 herbaria represented 37 states/provinces and 496 U.S. counties (Supporting information Appendix S1, Figure 1). Specimen collection years ranged from 1885–2015 (mean age = 41.66 years, ±26.25). Overall genotyping success was high, as data were obtained at all 15 loci in 59.9% (303) of samples and ≥12 loci in 86.8% (439) of samples. The generalized linear model demonstrated a significant effect of specimen age (*p* = <0.001) but not of herbarium of origin (*p* = 0.180) on the number of loci genotyped (Figures 2 and 3).

### 3.2 Isolation by distance

**Figure 4:**
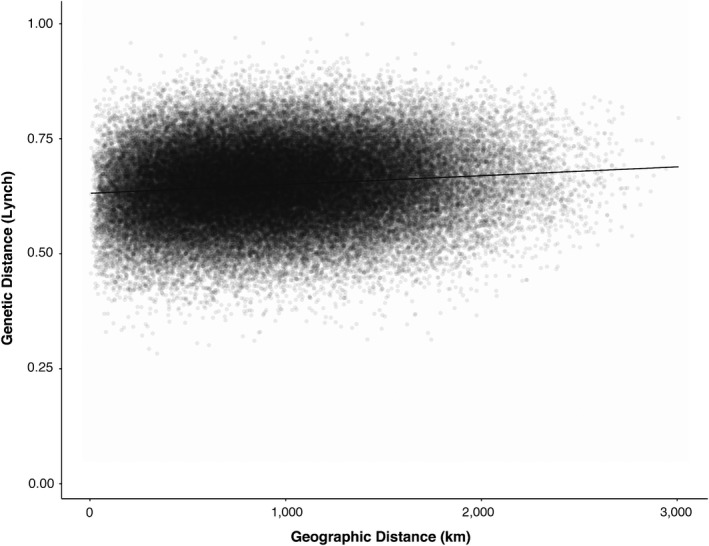
Scatterplot demonstrating the relationship between geographic and genetic distances among 439 samples from which ≥12‐loci were amplified. The genetic and geographic distance matrices are correlated (Mantel test: *p* = 0.001, *R* ^2^ = 0.013). A linear regression line has been added for visualization

All Mantel tests (≥10, ≥12, and ≥14‐locus datasets) were significant (*p* = 0.001, *R* ^2^ = 0.015; *p* = 0.001, *R* ^2^ = 0.013; *p* = 0.001, *R* ^2^ = 0.016, respectively), as were all such tests ≥12‐locus dataset subsets, (“old”: *p* = 0.001, *R* ^2^ = 0.011; “young”: *p* = 0.001, *R* ^2^ = 0.016; “quality”: *p* = 0.003, *R* ^2^ = 0.012) (Figure 4). The Mantel correlogram analysis established that 10/15 distance classes were significant (Table 2). The Evanno et al. (2005) summary of the STRUCTURE analysis identified an optimal *K* of 4 for both the diploid‐only and diploidized versions of the ≥12‐locus dataset. However, at *K* = 4, all samples exhibited nearly equal cluster assignments. In the diploid‐only analysis, only 8.66% (38 samples) exhibited a cluster assignment of ±25%, while all other samples exhibited an assignment of exactly 25% to each cluster. In the diploidized analysis, only 18% (79 samples) had a cluster assignment of ±25%.

**Table 2:** Mantel correlogram results

Distance class midpoint (m)	Mantel correlation	Corrected *p*‐value
300	0.0581	0.001***
500	0.0313	0.002**
700	0.0154	0.008**
900	−0.004	0.208
1,100	−0.0183	0.005**
1,300	−0.0282	0.006**
1,500	−0.0372	0.007**
1,700	−0.0386	0.008**
1,900	−0.027	0.009**
2,100	−0.0264	0.01**
2,300	−0.0269	0.011*
2,500	−0.0098	0.134
2,700	0.0103	0.138
2,900	−0.0037	0.34
3,100	−0.0058	0.15

*Note.* Significance ***p < 0.001, **p < 0.01, *p < 0.05.

Mantel correlation refers to the Pearson correlation obtained from the R package “vegan.”

## 4 DISCUSSION

A significant but weak signal of IBD was found across the *P. serotina* var. *serotina* range, regardless of dataset type and largely regardless of distance class. The fact that similar levels of IBD were observed in the total ≥12‐locus, “young” ≥12‐locus, and “old” ≥12‐locus datasets suggests that IBD has not changed qualitatively in this species over the past century, and that the modest signal we observed is not an artifact of comparing samples representing different collection periods. Similarly, the signal observed with the “quality” ≥12‐locus dataset suggests that low observed IBD is not a product of over‐sampling individuals from anthropogenic sites. Herbarium collections are strongly biased toward roadsides and other human‐accessible areas (Daru et al., 2018), a fact that should be considered when choosing specimens for landscape genetic study. Consistent with this modest relationship between genetic similarity and geographic distance, STRUCTURE suggests that few samples are strongly assigned to any of the most optimal set of genetic groups. It is important to emphasize that our dataset is different from most studies in that we utilized a “many populations, one individual per population” (inter‐individual) strategy, as opposed to a one featuring a “few populations, many individuals per population” (inter‐populational) strategy. The standard inter‐populational dataset considers variation among individuals within sites, but leads to a sparse and often geographically uneven array of sites. Our inter‐individual strategy instead emphasizes a denser and more geographically even array of sites, but does not account for within‐site variation. We are therefore calculating inter‐individual genetic/geographic distances rather than inter‐population distances. Isolation by Distance is of course a hypothesis regarding the genetic/geographic relationship between populations, and our assessment of these relationships among individuals is only valid if these individuals are representative of their home populations. If gene flow is indeed strong relative to genetic drift among eastern black cherry populations (as our data strongly suggest), the potential for comparing non‐representative genotypes increases. Eastern black cherry populations harbor considerable within‐population neutral genetic variation. The seven populations surveyed in Beck et al. (2014) exhibited an average observed heterozygosity of 0.75 across at SSR loci, and the 22 populations surveyed by Pairon et al. (2010) exhibited an average observed heterozygosity of 0.72 at eight SSR loci. Both values are higher than the average (0.65) reported for the 71 outcrossing plant taxa reviewed in Nybom (2004).

Indeed, two recent meta‐analyses suggest that a strategy of comparing inter‐individual genetic/geographic distances decreases the expectation of observing IBD. Jenkins et al. (2010) reviewed 240 studies that featured the typical inter‐population strategy. They established that the number of populations sampled does strongly influence the probability of a significant Mantel test *p*‐value, suggesting that those featuring >30 populations should obtain this IBD signal in essentially all cases (Figure 2, that study). Interestingly however, the strength of the genetic/geographic correlation (*R* ^2^) fell with population number, perhaps due to the increased landscape complexity sampled with a larger population matrix. In contrast, Pelletier and Carstens (2018) examined IBD in 8,955 species by comparing inter‐individual distances. As in Jenkins et al. (2010), the probability of observing a significant IBD Mantel test increased with sample size, even at large sample sizes (>150 individuals) only ca. 40% of studies are expected to observe IBD (Pelletier & Carstens, 2018: fig. S3). Future simulation studies should explore the role that sampling intensity and strategy play in the detection of genetic structure (Prunier et al., 2013).

Mantel tests establish that only 1%–2% of the variation in genetic distances among eastern black cherry individuals can be explained by straight‐line geographic distance. For comparison, geographic distance on average explained 22% of such variation in the 240 inter‐population studies reviewed in Jenkins et al. (2010). Although the only other study to examine IBD in North American *Prunus serotina* s.l. (Guzman, Segura, Aradhya, & Potter, 2018) reported a considerably higher value (*R* ^2^ = 0.45), this study differed from ours in critical respects. Besides featuring an inter‐population sampling strategy, their 18 populations spanned the four *Prunus serotina* s.l. varieties found in Mexico and Texas. Their measure of IBD therefore combines geographic limits to gene flow due to both distance and potential reproductive incompatibility among varieties.

The apparently powerful homogenizing force of gene flow across *P. serotina* var. *serotina*'s range is consistent with its life history. The species is fast‐growing (Auclair, 1975), pollinated by generalist insects (Fortuna, García, Guimarães, & Bascompte, 2008; Guitian, Guitian, & Sanchez, 1993; Jacobs et al., 2009; Lander et al., 2013), and has seeds that are potentially dispersed via ornithochory (Marks, 1974; Thompson & Willson, 1979). It also is often found in ruderal habitats, many of which are likely dispersal corridors (road margins, fencerows, railroads). Indeed, *P. serotina* var. *serotina* has become a highly problematic invasive species in Europe (Pairon et al., 2010). The Floristic Quality Assessment (FQA) method incorporates a coefficient of conservatism assigned to each species based on its tolerance of anthropogenic disturbance, with these “*C*‐values” ranging from 0 (high tolerance) to 10 (low tolerance) (Freyman, Masters, & Packard, 2016). Although *C*‐values are partially subjective, they have been shown to consistently track aspects of plant life history and community membership (Bauer, Koziol, & Bever, 2018; Matthews, Spyreas, & Long, 2014). *C*‐values are assigned by expert botanists on a state or regional level, and those reported below are averages of at least three scores per taxon from McAvoy (2018) (Delaware), Reznicek, Penskar, Walters, and Slaughter (2014) (Michigan), Ladd and Thomas (2015) (Missouri), Andreas, Mack, and McCormac (2004) (Ohio), Gianopulos (2014) (Southeastern US wetlands), or the Mid‐Atlantic Wetland Workgroup (2018). *Prunus serotina* var. *serotina* received an average C‐value of 2.6, indicating little conservatism in habitat preference. For context, other eastern North American tree species with conservatism scores <4 include *Juniperus virginiana* L. (red cedar) (*C* = 2.8) and *Acer saccharinum* L. (silver maple) (*C* = 3.5). Both are commonly found in disturbed sites. In contrast, the eastern North American tree species displaying significant inter‐populational IBD discussed above receive higher *C*‐values (*Juglans nigra*,* C* = 4.7;* Juglans cinerea*,* C* = 6.6; *Cornus florida C* = 5.8) (Hadziabdic, 2010; Hoban et al., 2010; Victory et al., 2006).

### 4.1 Implications and future directions

If the low level of IBD observed here indeed results from eastern black cherry's life history, this result requires context before any generalization is made regarding widespread North American forest trees. Fortunately, similar rangewide inter‐individual studies of widespread woody plants typical of higher quality habitats could be easily undertaken given the efficiency of the herbarium sampling strategy and the availability of microsatellite markers. For example, a set of loci developed for *Liriodendron tulipifera* L. (tulip tree, *C* = 6.5, loci from Xu, Li, & Zhang, 2006) is available. Other candidate species include *Aesculus glabra* Willd. (American buckeye, *C* = 5.8, Minami, Isagi, Kaneko, & Kawaguchi, 1998)*; Cornus florida* L. (flowering dogwood *C* = 5.8, Wang et al., 2008); *Quercus rubra* L. (red oak *C* = 5.5, Aldrich, Michler, Sun, & Romero‐Severson, 2002); *Lindera benzoin* (L.) Blume (spice bush *C* = 5.8, Edwards & Niesenbaum, 2007), and *Cercis canadensis* L. (redbud *C* = 5.8, Wadl, Trigiano, Werner, Pooler, & Rinehart, 2012). These studies would lend context to the modest level of IBD in *P. serotina* var. *serotina* and help shape our understanding of gene flow in North American forests.

## CONFLICT OF INTEREST

The authors have identified no potential conflict of interests.

## AUTHOR CONTRIBUTIONS

J.B. and J.S. conceived the ideas; L.K. and J.B. collected the data; L.K. and J.B. analyzed the data; and L.K. and J.B. led the writing.

## Data Availability

All microsatellite genotypes and specimen locality data can be found in Dryad: Genotypes and Locality: https://doi.org/10.5061/dryad.r1f08nt.
