# Supplementary material for: A rangewide herbarium‐derived dataset indicates high levels of gene flow in black cherry (Prunus serotina)
Source: Ecol Evol. 2019 Jan 8;9(3):975–85. doi: 10.1002/ece3.4719 (PMC6374653; doi:10.1002/ece3.4719)
Supplement: Supplementary file 1 [file ECE3-9-975-s001.docx]

*Journal of Biogeography*

**SUPPORTING INFORMATION**

A RANGEWIDE HERBARIUM-DERIVED DATSET INDICATES HIGH LEVELS OF GENE FLOW IN BLACK CHERRY (*PRUNUS SEROTINA*)

Lauren Konrade, Joey Shaw, and James Beck

**Appendix S1** Information for 506 *Prunus serotina* samples

**Table S1** Sampling data for 506 Prunus serotina samples

| **Extraction** | **State** | **County** | **Herbarium** | **Collector** | **Latitude** | **Longitude** | **Locus Count** | **Age** |
| --- | --- | --- | --- | --- | --- | --- | --- | --- |
| JB1980 | Alabama | Cherokee | MO | Kral 59974 | 34.39 | -85.63 | 14 | 41 |
| JB1981 | Alabama | Crenshaw | MO | Kral 39467 | 31.72 | -86.26 | 15 | 48 |
| JB1982 | Alabama | Dale | MO | MacDonald 10239 | 31.27 | -85.62 | 15 | 21 |
| JB1983 | Alabama | Randolph | MO | Nixon 1287 | 33.15 | -85.62 | 9 | 113 |
| JB1984 | Alabama | Talladega | MO | Kral 46062 | 33.14 | -86.25 | 15 | 46 |
| JB1985 | Arkansas | Franklin | MO | Thompson C0328 | 35.25 | -94.14 | 15 | 29 |
| JB1986 | Arkansas | Searcy | MO | Demaree 70803 | 35.99 | -92.72 | 13 | 42 |
| JB1987 | Florida | Hillsborough | MO | Lakela 31336 | 27.95 | -82.46 | 15 | 50 |
| JB1988 | Florida | Jackson | MO | Hess 8496 | 30.75 | -84.92 | 15 | 19 |
| JB1989 | Florida | Okaloosa | MO | Miller 9482 | 30.57 | -86.55 | 15 | 20 |
| JB1990 | Georgia | Brooks | MO | McCarty s.n. | 30.79 | -83.71 | 12 | 45 |
| JB1991 | Georgia | Meriwether | MO | McVaugh 8942 | 32.94 | -84.58 | 15 | 70 |
| JB1992 | Georgia | Stephens | MO | Spongberg 1789 | 34.63 | -83.32 | 15 | 36 |
| JB1993 | Illinois | Du Page | MO | Hess 7869 | 41.77 | -88.05 | 15 | 21 |
| JB1994 | Indiana | Fulton | MO | Nee 55473 | 41.04 | -86.03 | 15 | 11 |
| JB1995 | Iowa | Palo Alto | MO | Hayden 8513 | 43.16 | -94.9 | 13 | 77 |
| JB1996 | Kentucky | Woodford | MO | Semple 2592 | 38.16 | -84.68 | 13 | 41 |
| JB1997 | Minnesota | Anoka | MO | Smith 28908 | 45.2 | -93.21 | 15 | 18 |
| JB1998 | Minnesota | Goodhue | MO | Smith 28896 | 44.53 | -92.34 | 14 | 18 |
| JB1999 | Minnesota | Otter Tail | MO | Smith 27375 | 46.63 | -95.31 | 15 | 20 |
| JB2000 | Minnesota | Ramsey | MO | Smith 29018 | 45.06 | -93.12 | 15 | 18 |
| JB2001 | Minnesota | Sherburne | MO | Smith 29046 | 45.5 | -93.65 | 15 | 18 |
| JB2002 | Minnesota | Washington | MO | Smith 27957 | 45.22 | -92.77 | 15 | 19 |
| JB2003 | Mississippi | Forrest | MO | McDaniel 31439 | 31.22 | -89.17 | 14 | 26 |
| JB2004 | Missouri | Audrain | MO | Moe 05-19 | 39.07 | -91.64 | 15 | 13 |
| JB2005 | Missouri | Barry | MO | Hornberger 174 | 36.59 | -93.83 | 13 | 39 |
| JB2006 | Missouri | Barton | MO | Timme 14198 | 37.3 | -94.38 | 15 | 21 |
| JB2007 | Missouri | Butler | MO | Bornstein 463 | 36.89 | -90.3 | 14 | 23 |
| JB2008 | Missouri | Cape Girardeau | MO | Priest 22 | 37.32 | -89.57 | 15 | 12 |
| JB2009 | Missouri | Crawford | MO | Yatskievych 93-17 | 37.94 | -91.21 | 14 | 25 |
| JB2010 | Missouri | Dent | MO | Nee 27326 | 37.63 | -91.26 | 14 | 35 |
| JB2011 | Missouri | Harrison | MO | Summers 8756B | 40.51 | -93.82 | 15 | 20 |
| JB2012 | Missouri | Lawrence | MO | Gibson 3537 | 37.12 | -93.58 | 15 | 16 |
| JB2013 | Missouri | Marion | MO | Davis 1225 | 39.71 | -91.36 | 14 | 102 |
| JB2014 | Missouri | Monroe | MO | Hinterthuer 396 | 39.52 | -91.72 | 12 | 45 |
| JB2015 | Missouri | Pettis | MO | McCauley 251 | 38.57 | -93.3 | 15 | 20 |
| JB2016 | Missouri | Pulaski | MO | Ovrebo W1331 | 37.63 | -92.17 | 12 | 29 |
| JB2017 | Missouri | Ralls | MO | Summers 8168 | 39.67 | -91.66 | 14 | 21 |
| JB2018 | Missouri | Randolph | MO | Pelton s.n. | 39.26 | -92.45 | 15 | 32 |
| JB2019 | Missouri | Reynolds | MO | Ladd 13687 | 37.42 | -91.09 | 14 | 29 |
| JB2020 | Missouri | Ripley | MO | Rowland 12 | 36.68 | -90.83 | 15 | 8 |
| JB2021 | Missouri | St Francois | MO | Darigo 1341 | 37.87 | -90.59 | 13 | 25 |
| JB2022 | Missouri | St Louis | MO | Feltz 80 | 38.54 | -90.54 | 15 | 9 |
| JB2023 | Missouri | Scott | MO | Powell 8 | 37.62 | -89.48 | 15 | 8 |
| JB2024 | Missouri | Texas | MO | Freeman 21499 | 37.33 | -91.95 | 15 | 12 |
| JB2025 | Nebraska | Nemaha | MO | Churchill 5125 | 40.45 | -95.71 | 13 | 43 |
| JB2026 | New Hampshire | Belknap | MO | Bradley 1432 | 43.61 | -71.4 | 15 | 14 |
| JB2027 | North Carolina | Avery | MO | Solomon 3989 | 36.08 | -81.78 | 15 | 40 |
| JB2028 | North Carolina | Graham | MO | Miller 8956 | 35.14 | -83.4 | 14 | 21 |
| JB2029 | North Carolina | Watauga | MO | Crosby 17769 | 36.28 | -81.84 | 15 | 17 |
| JB2030 | Oklahoma | Sequoyah | MO | Little 36195 | 35.41 | -94.6 | 15 | 38 |
| JB2031 | Ontario | York | MO | Soper 4614 | 43.83 | -79.2 | 12 | 68 |
| JB2032 | Pennsylvania | Adams | MO | Myers 20 | 39.71 | -77.4 | 12 | 28 |
| JB2034 | Quebec |  | MO | Roy 3188 | 45.53 | -73.7 | 8 | 84 |
| JB2035 | Vermont | Caledonia | MO | Seymour 21435 | 44.54 | -71.9 | 14 | 55 |
| JB2036 | Vermont | Orleans | MO | Seymour 25170 | 44.91 | -71.98 | 15 | 52 |
| JB2037 | Wisconsin | Richland | MO | Nee 14503B | 43.3 | -90.33 | 15 | 42 |
| JB2038 | Wisconsin | Vernon | MO | Ziegler 1746 | 43.7 | -91.26 | 11 | 43 |
| JB2103 | Alabama | Autauga | VDB | Haynes 9220 | 32.69 | -86.73 | 15 | 31 |
| JB2104 | Alabama | Calhoun | VDB | Hruska 829 | 33.59 | -85.88 | 15 | 23 |
| JB2105 | Alabama | Coffee | VDB | Kral 89583 | 31.3 | -86.14 | 15 | 18 |
| JB2106 | Alabama | Covington | VDB | Diamond 13015 | 31.44 | -86.26 | 15 | 16 |
| JB2107 | Alabama | De Kalb | VDB | Spaulding 5014 | 34.46 | -86.05 | 15 | 25 |
| JB2108 | Alabama | Etowah | VDB | Kral 92815 | 34.1 | -85.99 | 15 | 16 |
| JB2109 | Alabama | Franklin | VDB | Kral 26304 | 34.37 | -87.75 | 15 | 52 |
| JB2110 | Alabama | Henry | VDB | Diamond 19107 | 31.55 | -85.36 | 14 | 10 |
| JB2111 | Alabama | Houston | VDB | MacDonald 7638 | 31.24 | -85.51 | 15 | 24 |
| JB2112 | Alabama | Jackson | VDB | DiPietro s.n. | 34.98 | -85.81 | 15 | 25 |
| JB2113 | Alabama | Jefferson | VDB | Williams 66 | 33.42 | -86.73 | 15 | 55 |
| JB2114 | Alabama | Lee | VDB | Kral 30769 | 32.52 | -85.25 | 15 | 50 |
| JB2115 | Alabama | Lowndes | VDB | Diamond 12961 | 32.33 | -86.51 | 14 | 16 |
| JB2116 | Alabama | Macon | VDB | Diamond 13009 | 32.4 | -85.99 | 15 | 16 |
| JB2117 | Alabama | Madison | VDB | Kral 43342 | 34.96 | -86.37 | 15 | 47 |
| JB2118 | Alabama | Perry | VDB | Kral 66736 | 32.78 | -87.28 | 15 | 37 |
| JB2119 | Alabama | Pickens | VDB | Kral 45265 | 33.11 | -87.92 | 15 | 46 |
| JB2120 | Alabama | Pike | VDB | Diamond 13803 | 31.91 | -86.06 | 15 | 15 |
| JB2121 | Alabama | Russell | VDB | Diamond 16283 | 32.34 | -85.41 | 15 | 12 |
| JB2122 | Alabama | St Clair | VDB | Keener 4419 | 33.95 | -86.28 | 15 | 10 |
| JB2123 | Alabama | Sumter | VDB | Jones 15560 | 32.57 | -88.3 | 15 | 50 |
| JB2124 | Alabama | Washington | VDB | Kral 37349B | 31.2 | -88.01 | 15 | 49 |
| JB2125 | Alabama | Winston | VDB | Kral 23734 | 34.11 | -87.62 | 15 | 53 |
| JB2126 | Tennessee | Coffee | VDB | Blum 3517 | 35.41 | -86.12 | 15 | 49 |
| JB2127 | Tennessee | Cumberland | VDB | Shaffer 91 | 35.96 | -85.13 | 15 | 39 |
| JB2128 | Tennessee | Davidson | VDB | Kral 82613 | 36.1 | -86.82 | 15 | 25 |
| JB2129 | Tennessee | Dickson | VDB | Souza 86-651 | 36.09 | -87.27 | 15 | 32 |
| JB2130 | Tennessee | Franklin | VDB | Kral 30266 | 35.16 | -86.15 | 15 | 50 |
| JB2131 | Tennessee | Grundy | VDB | Patrick 353 | 35.46 | -85.62 | 15 | 41 |
| JB2132 | Tennessee | Hardin | VDB | Jones 2313 | 35.22 | -88.31 | 15 | 38 |
| JB2133 | Tennessee | Hickman | VDB | Estes 4711 | 35.73 | -87.63 | 15 | 15 |
| JB2134 | Tennessee | Humphreys | VDB | Kral 76313 | 36.03 | -87.83 | 15 | 29 |
| JB2135 | Tennessee | Lauderdale | VDB | Keiran 300 | 35.69 | -89.63 | 15 | 46 |
| JB2136 | Tennessee | Lewis | VDB | Howell 518 | 35.47 | -87.48 | 15 | 27 |
| JB2137 | Tennessee | Overton | VDB | Kral 82203 | 36.35 | -85.33 | 15 | 25 |
| JB2138 | Tennessee | Robertson | VDB | Blum 2910 | 36.55 | -87.11 | 15 | 50 |
| JB2139 | Tennessee | Sumner | VDB | Alcorn 291 | 36.54 | -86.47 | 15 | 43 |
| JB2140 | Tennessee | Wayne | VDB | Kral 31670 | 35.33 | -87.64 | 15 | 50 |
| JB2141 | Texas | Cass | SMU | McVaugh 7164 | 33.3 | -94.14 | 15 | 73 |
| JB2142 | Texas | Cherokee | BRIT | Ajilvsgi 5071 | 32.11 | -95.05 | 15 | 41 |
| JB2143 | Texas | Hardin | SMU | Cory 54921 | 30.25 | -94.18 | 13 | 70 |
| JB2144 | Texas | Harris | SMU | Traverse 1317 | 29.8 | -95.52 | 15 | 59 |
| JB2145 | Texas | Jasper | SMU | Cory 52743 | 30.38 | -93.9 | 12 | 71 |
| JB2146 | Texas | Kaufman | SMU | Shinners 15414 | 32.41 | -96.23 | 7 | 65 |
| JB2147 | Texas | Lamar | SMU | McVaugh 7146 | 33.66 | -95.6 | 13 | 73 |
| JB2148 | Texas | Leon | BRIT | Nixon 17652 | 29.95 | -96.75 | 15 | 26 |
| JB2149 | Texas | Montgomery | BRIT | Sanders 6015 | 30.13 | -95.18 | 15 | 15 |
| JB2150 | Texas | Nacogdoches | SMU | McVaugh 8407 | 31.53 | -94.39 | 15 | 71 |
| JB2151 | Texas | Newton | SMU | McVaugh 6860 | 30.85 | -93.82 | 15 | 73 |
| JB2152 | Texas | Red River | BRIT | Sanders 2040 | 33.45 | 95.05 | 12 | 25 |
| JB2153 | Texas | Titus | SMU | Amerson 902 | 33.13 | -95.1 | 12 | 47 |
| JB2154 | Texas | Wood | BRIT | Wagnon 129 | 32.89 | -95.23 | 15 | 18 |
| JH086 | Wisconsin | Richland | WIS | Nee 24267 | 43.31 | -90.31 | 0 | 36 |
| JH087 | Wisconsin | Waukesha | WIS | Leitner 3714 | 43.03 | -88.3 | 0 | 29 |
| JH088 | Michigan | Cheboygan | MICH | McVaugh10875 | 45.56 | -84.67 | 0 | 69 |
| JH264 | Georgia | Seminole | FLAS | Gholson 3003 | 30.78 | -84.87 | 9 | 48 |
| JH265 | Florida | Gadsden | FLAS | Gholson 9498 | 30.71 | -84.85 | 6 | 36 |
| JH266 | Florida | Nassau | FLAS | Rider 180 | 30.77 | -81.73 | 15 | 18 |
| JH267 | Virginia | Prince William | FLAS | Keyser 708 | 38.66 | -77.25 | 12 | 36 |
| JH268 | Kentucky | Madison | FLAS | Abbott 2134 | 37.9 | -84.27 | 15 | 26 |
| JH269 | Virginia | York | FLAS | Kirkman 201 | 37.29 | -76.6 | 14 | 43 |
| JH270 | West Virginia | Upshur | FLAS | Rossbach 7255 | 39 | -80.21 | 1 | 52 |
| JH271 | West Virginia | Randolph | FLAS | Clendening s.n. | 38.85 | -79.56 | 11 | 54 |
| JH272 | Virginia | Accomack | FLAS | Ware 6654 | 37.62 | -75.69 | 15 | 41 |
| JH273 | South Carolina | Richland | FLAS | Nelson 514 | 34.04 | -80.98 | 15 | 42 |
| JH274 | Louisiana | West Feliciene | FLAS | Urbatsch 2285 | 30.75 | -91.29 | 13 | 42 |
| JH275 | Louisiana | Temmeny | FLAS | Rylander 35 | 30.48 | -90.1 | 15 | 55 |
| JH276 | Louisiana | Ouchita | FLAS | Thomas 22580 | 32.52 | -92.1 | 14 | 47 |
| JH277 | Florida | Jackson | FLAS | Hess 8496 | 30.75 | -84.92 | 8 | 19 |
| JH278 | Florida | Hillsborough | FLAS | Lakela 26099 | 28 | 82.46 | 15 | 55 |
| JH280 | Florida | Levy | FLAS | Golledge 525 | 29.5 | -82.97 | 15 | 113 |
| JH281 | Florida | Columbia | FLAS | Tan 411 | 29.91 | -82.58 | 15 | 28 |
| JH282 | Florida | Clay | FLAS | Ferguson 62 | 29.78 | -82.01 | 15 | 22 |
| JH283 | Florida | Alachua | FLAS | Lange 1396 | 29.65 | -82.35 | 15 | 6 |
| JH284 | Florida | Walton | FLAS | Perkins 16401 | 30.46 | -81.42 | 15 | 41 |
| JH285 | Florida | Suwanee | FLAS | Herring 291 | 29.95 | -82.79 | 15 | 27 |
| JH286 | Florida | Polk | FLAS | Conard s.n. | 28.04 | -81.64 | 15 | 52 |
| JH287 | Florida | Liberty | FLAS | Sloan 1612 | 30.55 | -84.94 | 15 | 33 |
| JH288 | Florida | Marion | FLAS | George 23 | 28.98 | -81.9 | 15 | 13 |
| LK1000 | Arkansas | Garland | KANU | Demaree 36747 | 34.51 | -93.08 | 14 | 63 |
| LK1001 | Indiana | Blackford | BUT | Friesner 10942 | 40.54 | -85.38 | 15 | 81 |
| LK1002 | Indiana | Brown | BUT | RCF s.n. | 39.26 | -86.34 | 14 | 88 |
| LK1003 | Indiana | Dubois | BUT | Friesner 5177 | 38.31 | -86.84 | 15 | 86 |
| LK1004 | Indiana | Fountain | BUT | Friesner 3973 | 40.06 | -87.34 | 7 | 86 |
| LK1005 | Indiana | Harrison | BUT | Friesner 2940 | 38.3 | -86.1 | 15 | 87 |
| LK1006 | Indiana | Kosciusko | BUT | Friesner 13437 | 41.07 | -85.96 | 12 | 79 |
| LK1007 | Indiana | Lawrence | BUT | Friesner 4941 | 38.85 | -86.53 | 13 | 86 |
| LK1008 | Indiana | Madison | BUT | Rothrock 2110 | 40.1 | -85.62 | 15 | 28 |
| LK1009 | Indiana | Marion | BUT | Crandall 43 | 39.87 | -86.19 | 15 | 22 |
| LK1010 | Indiana | Miami | BUT | Friesner 15478 | 40.87 | -86.14 | 15 | 77 |
| LK1011 | Indiana | Monroe | BUT | Friesner 3184 | 39.06 | -86.4 | 15 | 87 |
| LK1012 | Indiana | Parke | BUT | Daubenmire 2296 | 39.76 | -87.23 | 3 | 88 |
| LK1013 | Indiana | St. Joseph | BUT | Friesner 13560 | 41.53 | -86.28 | 15 | 79 |
| LK1014 | Indiana | Tipton | BUT | Friesner 11730 | 40.29 | -86.17 | 14 | 81 |
| LK1015 | Indiana | Wabash | BUT | Friesner 15290 | 40.68 | -85.83 | 14 | 77 |
| LK1016 | Indiana | Warren | BUT | Tonkovich 29 | 40.34 | -87.32 | 15 | 27 |
| LK1017 | Indiana | Washington | BUT | Friesner 5007 | 38.65 | -86.1 | 15 | 86 |
| LK1018 | Indiana | White | BUT | Loughridge 1713 | 40.74 | -86.78 | 6 | 83 |
| LK1019 | Iowa | Adams | ISC | Isely 73 | 41.12 | -94.87 | 15 | 71 |
| LK1020 | Iowa | Clayton | ISC | Pauieel s.n. | 43.02 | -91.18 | 15 | 93 |
| LK1021 | Iowa | Davis | ISC | Aikman s.n. | 40.75 | -92.42 | 15 | 93 |
| LK1022 | Iowa | Delaware | ISC | LHP s.n. | 47.62 | -91.56 | 15 | 94 |
| LK1023 | Iowa | Des Moines | ISC | Pammel 958 | 40.83 | -91.12 | 15 | 93 |
| LK1024 | Iowa | Floyd | KANU | Freeman 22431 | 43.15 | -92.64 | 0 | 11 |
| LK1025 | Iowa | Franklin | ISC | Monson 3645 | 42.66 | -93.24 | 8 | 62 |
| LK1026 | Iowa | Hamilton | ISC | Thompson s.n. | 42.45 | -93.79 | 12 | 16 |
| LK1027 | Iowa | Hardin | KANU | Freeman 9738 | 42.45 | -93.38 | 14 | 21 |
| LK1028 | Iowa | Howard | ISC | Christiansen 861 | 43.44 | -92.38 | 15 | 40 |
| LK1029 | Iowa | Humbodt | ISC | Monson 3566 | 42.87 | -94.19 | 10 | 62 |
| LK1030 | Iowa | Lee | ISC | Fults 1219 | 40.46 | -91.4 | 12 | 87 |
| LK1031 | Iowa | Mahaska | ISC | Augustine 66 | 41.28 | -92.46 | 10 | 80 |
| LK1032 | Iowa | Page | ISC | Wilson 1047 | 42.49 | -98.32 | 15 | 31 |
| LK1033 | Iowa | Polk | ISC | Pammel 339 | 41.58 | -93.68 | 12 | 93 |
| LK1034 | Iowa | Story | ISC | Couch 016 | 42.03 | -93.65 | 10 | 31 |
| LK1035 | Iowa | Tama | ISC | LHP s.n. | 42.19 | -92.47 | 13 | 95 |
| LK1036 | Iowa | Warren | ISC | Croat 25009 | 41.35 | -93.76 | 14 | 45 |
| LK1037 | Iowa | Webster | ISC | Niemann 308 | 42.42 | -94.1 | 11 | 48 |
| LK1038 | Iowa | Winneshiek | ISC | Norris 9869101 | 43.4 | -91.9 | 15 | 20 |
| LK1039 | Kansas | Anderson | KANU | Morse 8197 | 38.27 | -95.25 | 12 | 16 |
| LK1040 | Kansas | Brown | KANU | McGregor 17069 | 39.85 | -95.37 | 14 | 57 |
| LK1041 | Kansas | Franklin | KANU | Freeman 12837 | 38.58 | -95.27 | 14 | 19 |
| LK1042 | Kansas | Jefferson | KANU | Stehpens 89120 | 39.32 | -95.46 | 8 | 42 |
| LK1043 | Kansas | Johnson | KANU | Freeman 9144 | 38.83 | -94.64 | 6 | 21 |
| LK1044 | Kansas | Kingman | KANU | Stephens 53505 | 37.66 | -98.21 | 15 | 46 |
| LK1045 | Michigan | Alcona | MICH | Garlitz 837 | 44.84 | -83.83 | 15 | 34 |
| LK1046 | Michigan | Alger | MICH | Freudenstein 1402 | 46.65 | -86.11 | 15 | 34 |
| LK1047 | Michigan | Antrim | MICH | Appel 232 | 45.11 | -85.1 | 14 | 38 |
| LK1048 | Michigan | Bay | MICH | Fruedenstein 1543 | 43.69 | -83.93 | 15 | 33 |
| LK1049 | Michigan | Crawford | MICH | Chittenden 478 | 44.71 | -84.59 | 15 | 26 |
| LK1050 | Michigan | Genesee | MICH | Merkle 70108 | 42.84 | -83.73 | 13 | 48 |
| LK1051 | Michigan | Gratiot | MICH | Freudenstein 847 | 43.26 | -84.4 | 15 | 35 |
| LK1052 | Michigan | Ionia | MICH | Gereau 984 | 42.84 | -84.87 | 15 | 36 |
| LK1053 | Michigan | Leelanau A | MICH | Hazlett 1826 | 45.1 | -86.04 | 14 | 36 |
| LK1054 | Michigan | Lenawee | MICH | Smith 530 | 41.91 | -84.04 | 15 | 33 |
| LK1055 | Michigan | Marquette | MICH | McVaugh 11133 | 46.54 | -87.52 | 15 | 69 |
| LK1056 | Michigan | Mason | MICH | Hazlett 854 | 44.08 | -86.33 | 1 | 39 |
| LK1057 | Michigan | Mecosta | MICH | Ross 1180 | 43.71 | -85.33 | 15 | 17 |
| LK1058 | Michigan | Missaukee | MICH | Voss 3084 | 44.51 | -84.92 | 12 | 62 |
| LK1059 | Michigan | Monroe | MICH | Easterly 11609 | 41.91 | -83.6 | 9 | 38 |
| LK1060 | Michigan | Montmorency | MICH | Garlitz 863 | 44.88 | -84.15 | 15 | 34 |
| LK1061 | Michigan | Ontonagon | MICH | MacFarlane 4895 | 46.77 | -89.08 | 0 | 32 |
| LK1062 | Michigan | Oscoda | MICH | Zimmerman 210 | 44.64 | -84.34 | 10 | 67 |
| LK1063 | Michigan | Saginaw | MICH | Freudenstein 1290 | 43.43 | -84.06 | 12 | 34 |
| LK1064 | Missouri | Chariton | KANU | Freeman 23002 | 39.38 | -93.02 | 15 | 10 |
| LK1066 | Oklahoma | Murray | KANU | Goodman 7935 | 34.44 | -97.02 | 0 | 49 |
| LK1067 | Wisconsin | Crawford | WIS | Moore 48 | 43.02 | -91.11 | 14 | 38 |
| LK1068 | Wisconsin | Dane | WIS | Cochrane 11860 | 43.21 | -89.73 | 15 | 29 |
| LK1069 | Wisconsin | Dodge | WIS | Leitner 1264 | 43.42 | -88.7 | 15 | 30 |
| LK1070 | Wisconsin | Door | WIS | Judziewicz 13743 | 45.19 | -87.36 | 6 | 18 |
| LK1071 | Wisconsin | Grant | WIS | Anderson 60 | 42.83 | -91.07 | 15 | 25 |
| LK1072 | Wisconsin | Green Lake | WIS | Banks 166 | 43.7 | -89.01 | 15 | 35 |
| LK1073 | Wisconsin | Iron | WIS | Lucy 6001 | 46.02 | -90.01 | 15 | 29 |
| LK1074 | Wisconsin | Juneau | WIS | Freckmann 24661 | 44.05 | -90.17 | 15 | 29 |
| LK1075 | Wisconsin | Kenosha | WIS | Smith 735 | 42.58 | -87.95 | 14 | 30 |
| LK1076 | Wisconsin | Manitowoc | WIS | Moore 532 | 44.28 | -87.76 | 14 | 32 |
| LK1077 | Wisconsin | Ozaukee | WIS | Leitner 3081 | 43.38 | -87.99 | 14 | 29 |
| LK1078 | Wisconsin | Sauk | WIS | Cochrane 11820 | 43.45 | -89.82 | 14 | 29 |
| LK1079 | Wisconsin | Sawyer | WIS | Weshinskey 237 | 46.11 | -91.31 | 13 | 25 |
| LK1080 | Wisconsin | Shawano | WIS | De Stefano 153 | 44.61 | -88.49 | 14 | 38 |
| LK1081 | Wisconsin | Taylor | WIS | Fields 1109 | 45.25 | -90.62 | 15 | 24 |
| LK1082 | Wisconsin | Walworth | WIS | Cochrane 12445 | 42.8 | -88.62 | 14 | 28 |
| LK1084 | Kentucky | Allen | KANU | Hulbert3783 | 37.91 | -95.11 | 12 | 59 |
| LK1085 | Kentucky | Franklin | KANU | McGregor10473 | 38.48 | -95.34 | 6 | 63 |
| LK1086 | Nebraska | Richardson | KANU | McGregor19102 | 40.14 | -95.72 | 12 | 53 |
| LK1089 | Florida | Gilchrist | NYBG | Longbottom 18664 | 29.82 | -82.69 | 15 | 5 |
| LK1090 | Indiana | Jasper | NYBG | Welsh 63 | 40.79 | -87.23 | 15 | 95 |
| LK1091 | Kentucky | Fleming | NYBG | Wharton 3823f | 38.33 | -83.56 | 6 | 79 |
| LK1092 | Kentucky | Henry | NYBG | Gentry 299 | 38.42 | -84.97 | 14 | 56 |
| LK1093 | Louisiana | Orleans | NYBG | Purrington 16 | 30 | -90.1 | 15 | 32 |
| LK1094 | Maine | Washington | NYBG | Atha 8807 | 44.68 | -67.95 | 14 | 8 |
| LK1095 | Maryland | Allegany | NYBG | Longbottom 11447 | 39.52 | -78.91 | 15 | 10 |
| LK1097 | Maryland | Howard | NYBG | Longbottom 13180 | 39.12 | -76.8 | 14 | 8 |
| LK1098 | Maryland | Queen Annes | NYBG | Longbottom 13227 | 38.97 | -76.13 | 15 | 8 |
| LK1099 | Maryland | Talbot | NYBG | Longbottom 13182 | 38.8 | -76.06 | 15 | 8 |
| LK1100 | Maryland | Washington | NYBG | Longbottom 11415 | 39.7 | -77.94 | 15 | 10 |
| LK1101 | Maryland | Wicomico | NYBG | Longbottom 11814 | 38.32 | -75.61 | 15 | 10 |
| LK1102 | Maryland | Worcester | NYBG | Hill 13899 | 38.24 | -75.14 | 15 | 34 |
| LK1104 | Massuchusetts | Dukes | NYBG | MacKeever 612 | 41.38 | -70.51 | 14 | 55 |
| LK1105 | New Jersey | Burlington | CHRB | Long 10697 | 39.59 | -74.45 | 15 | 4 |
| LK1108 | New Jersey | Essex | CHRB | Morton 6073 | 40.77 | -74.28 | 15 | 42 |
| LK1109 | New Jersey | Hunterdon | CHRB | Hough s.n. | 40.64 | -75.06 | 14 | 54 |
| LK1110 | New Jersey | Mercer | CHRB | Kramer 1341 | 40.38 | -74.65 | 15 | 52 |
| LK1112 | New Jersey | Morris | NYBG | Atha 10445 | 40.91 | -74.56 | 15 | 7 |
| LK1113 | New Jersey | Ocean | NYBG | Atha 6539 | 39.66 | -74.35 | 15 | 10 |
| LK1114 | New Jersey | Passaic | CHRB | Barringer 9664 | 40.98 | -74.32 | 15 | 15 |
| LK1115 | New Jersey | Salem | CHRB | Vhrysler 1130 | 39.71 | -75.35 | 7 | 83 |
| LK1116 | New Jersey | Somerset | CHRB | Costich 17 | 40.46 | -74.75 | 15 | 27 |
| LK1119 | New Jersey | Warren | CHRB | Hanks s.n. | 40.89 | -74.96 | 13 | 52 |
| LK1120 | New York | Bronx | NYBG | Nee 57867 | 40.87 | -73.89 | 15 | 7 |
| LK1121 | New York | Dutchess | CHRB | Ahles 66245 | 41.79 | -73.92 | 15 | 51 |
| LK1122 | New York | Putnam | NYBG | Atha 6543 | 41.39 | -73.91 | 15 | 10 |
| LK1123 | North Carolina | Cumberland | NYBG | Cruchfield 5612 | 35.06 | -78.96 | 15 | 50 |
| LK1124 | Ohio | Guernsey | NYBG | Nee 56389 | 40.12 | -81.56 | 15 | 9 |
| LK1125 | Pennsylvania | Armstrong | PH | Wahl 4798 | 40.88 | -79.58 | 8 | 71 |
| LK1126 | Pennsylvania | Blair | PH | Skinner 43 | 40.43 | -78.49 | 3 | 75 |
| LK1127 | Pennsylvania | Centre | PH | Fogg 17873 | 40.98 | -77.63 | 11 | 78 |
| LK1128 | Pennsylvania | Clarion | PH | Wahl 5586 | 41.02 | -79.38 | 15 | 70 |
| LK1129 | Pennsylvania | Clinton | PH | Wahl 2136 | 41.06 | -77.37 | 8 | 71 |
| LK1130 | Pennsylvania | Crawford | PH | Thompson 05721 | 41.7 | -80.42 | 15 | 13 |
| LK1131 | Pennsylvania | Erie | PH | Phillips 78123 | 41.99 | -80.21 | 15 | 99 |
| LK1132 | Pennsylvania | Franklin | PH | Wahl 6664 | 39.93 | -77.54 | 15 | 69 |
| LK1133 | Pennsylvania | Huntingdon | PH | Wahl 3549 | 40.33 | -78.02 | 14 | 71 |
| LK1134 | Pennsylvania | Indiana | PH | Skinner 100 | 40.66 | -79.02 | 9 | 76 |
| LK1135 | Pennsylvania | Jefferson | PH | Wahl 3633 | 41.13 | -79.18 | 13 | 71 |
| LK1137 | Pennsylvania | Luzerne | PH | Wahl 15489 | 41.36 | -76.04 | 10 | 63 |
| LK1138 | Pennsylvania | Monroe | PH | Wherry s.n. | 40.95 | -75.42 | 15 | 66 |
| LK1139 | Pennsylvania | Perry | CHRB | Adams 1405 | 40.43 | -77.2 | 12 | 84 |
| LK1140 | Pennsylvania | Pike | PH | DePue 403 | 41.17 | -74.91 | 15 | 80 |
| LK1141 | Pennsylvania | Potter | PH | Wahl 7930 | 41.87 | -77.72 | 14 | 69 |
| LK1142 | Pennsylvania | Schuylkill | PH | Wagner 4429 | 40.65 | -76.61 | 15 | 81 |
| LK1143 | Pennsylvania | Sullivan | NYBG | Penny 145 | 41.41 | -76.61 | 15 | 76 |
| LK1145 | Pennsylvania | Warren | PH | Wahl 11248 | 41.73 | -79.06 | 13 | 67 |
| LK1146 | Pennsylvania | Wayne | PH | Harper 2186 | 41.24 | -75.38 | 15 | 72 |
| LK1149 | Virgina | Northampton | CHRB | Small s.n. | 37.16 | -75.98 | 14 | 83 |
| LK1150 | Virginia | Washington | NYBG | Britton s.n. | 36.64 | -81.61 | 0 | 126 |
| LK1151 | West Virginia | Grant | NYBG | Atha 6627 | 39.06 | -79.28 | 15 | 10 |
| LK1152 | West Virginia | Wayne | CHRB | Gilbert 486 | 38.32 | -82.5 | 15 | 82 |
| LK1153 | Ontario | Leeds | NYBG | Dore 19936 | 44.36 | -76.01 | 15 | 56 |
| LK1154 | Quebec | Gatineau | NYBG | Cody 12375 | 45.55 | -76.09 | 15 | 56 |
| LK1209 | Arkansas | Arkansas | STAR | Richards 9516 | 34.37 | -91.12 | 10 | 33 |
| LK1210 | Arkansas | Baxter | STAR | Richards 9339 | 36.34 | -92.47 | 11 | 33 |
| LK1211 | Georgia | Bulloch | USF | Hill 27643 | 32.4 | -81.76 | 12 | 22 |
| LK1212 | Arkansas | Carroll | STAR | Atkins 131 | 36.33 | -93.39 | 15 | 34 |
| LK1213 | Arkansas | Clay | STAR | Hitt 2 | 36.39 | -90.2 | 11 | 52 |
| LK1214 | Arkansas | Cleburne | STAR | Babb 751 | 35.5 | -92.22 | 15 | 6 |
| LK1215 | Arkansas | Conway | STAR | Richards 10747 | 35.13 | -92.73 | 15 | 28 |
| LK1216 | Arkansas | Craighead | STAR | Scarborough 2 | 35.85 | -90.68 | 15 | 53 |
| LK1217 | Arkansas | Dallas | STAR | Richards 9665 | 34.14 | -92.67 | 15 | 33 |
| LK1218 | Arkansas | Drew | STAR | Richards 9299 | 33.61 | -91.81 | 15 | 33 |
| LK1235 | Mississippi | Adams | MISS | Temple 11259 | 31.51 | -91.37 | 14 | 49 |
| LK1236 | Mississippi | Alcorn | MISS | Pullen 66519 | 34.82 | -88.72 | 14 | 52 |
| LK1237 | Mississippi | Attala | MISS | Jones 16960 | 32.99 | -89.56 | 15 | 49 |
| LK1238 | Mississippi | Benton | MISS | Temple 5649 | 34.64 | -89.3 | 15 | 51 |
| LK1239 | Mississippi | Chickasaw | MISS | Pullen 66888 | 34.04 | -88.97 | 14 | 52 |
| LK1240 | Mississippi | Copiah | MISS | Herrington 014 | 31.87 | -90.5 | 15 | 54 |
| LK1241 | Mississippi | DeSoto | MISS | Ferrari 456 | 34.85 | -89.77 | 14 | 49 |
| LK1242 | Mississippi | George | MISS | Jones 6993 | 30.96 | -88.61 | 15 | 52 |
| LK1243 | Mississippi | Hinds | MISS | Temple 8099 | 32.35 | -90.39 | 11 | 50 |
| LK1244 | Mississippi | Jasper | MISS | Jones 13007 | 31.83 | -89.02 | 15 | 51 |
| LK1245 | Mississippi | Lafeyette | MISS | Connolly 66 | 34.43 | -89.39 | 10 | 18 |
| LK1246 | Mississippi | Lauderdale | MISS | Jones 7072 | 32.34 | -88.71 | 12 | 52 |
| LK1247 | Mississippi | Lawrence | MISS | Jones 7747 | 31.57 | -90.1 | 15 | 52 |
| LK1248 | Mississippi | Leflore | MISS | Bryson 17226 | 34.41 | -90.34 | 15 | 19 |
| LK1250 | South Carolina | Abbeville | NYBG | Credle 1164 | 34.18 | -82.69 | 15 | 39 |
| LK1251 | Illinois | Knox | NYBG | Chase 10626 | 40.93 | -90.01 | 7 | 4 |
| LK1252 | Illinois | Jo Daviess | NYBG | Nee 22011 | 42.26 | -90.29 | 15 | 37 |
| LK1253 | South Carolina | Richland | NYBG | Nelson 17928 | 34 | -81.4 | 15 | 21 |
| LK1254 | South Carolina | Aiken | NYBG | Hill 22373 | 33.6 | -81.84 | 15 | 27 |
| LK1255 | Virginia | Virginia Beach City | NYBG | Egler 40-108 | 36.88 | -76.1 | 15 | 78 |
| LK1256 | New York | Suffolk | NYBG | Pace 252 | 40.83 | -73.43 | 15 | 10 |
| LK1257 | North Carolina | Beaufort | NYBG | Atha 9983 | 35.31 | -76.8 | 15 | 7 |
| LK1258 | New York | Westchester | NYBG | Doody s.n. | 41.13 | -73.79 | 14 | 60 |
| LK1260 | New York | Franklin | NYBG | Britton s.n. | 44.56 | -74.33 | 15 | 118 |
| LK1261 | Virginia | Arlington | NYBG | Harriman s.n. | 38.89 | -77.07 | 15 | 24 |
| LK1262 | Ontario | La Haute-Yamaska | NYBG | Habuis 612 | 45.43 | -72.7 | 13 | 72 |
| LK1263 | Ontario | Kingston | NYBG | Fowler s.n. | 44.24 | -76.61 | 1 | 116 |
| LK1264 | Kentucky | Harlan | NYBG | Kearney s.n. | 36.81 | -83.31 | 15 | 125 |
| LK1265 | Georgia | De Kalb | NYBG | Small s.n. | 33.81 | -84.15 | 13 | 123 |
| LK1266 | Illinois | Adams | NYBG | Evers 61 | 39.87 | -91.3 | 2 | 79 |
| LK1267 | Rhode Island | Providence | HUH | Robinson s.n. | 41.97 | -71.43 | 14 | 114 |
| LK1268 | Connecticut | Tolland | HUH | Mehrhoff 14329 | 41.99 | -72.41 | 15 | 27 |
| LK1269 | Vermont | Chittenden | HUH | Cook 480 | 44.56 | -72.94 | 15 | 60 |
| LK1270 | Vermont | Windham | HUH | Wheeler s.n. | 43.04 | -72.66 | 15 | 106 |
| LK1271 | Connecticut | Fairfield | HUH | Green 67/63 | 41.02 | -73.63 | 10 | 55 |
| LK1272 | New Hampshire | Cheshire | HUH | Batchelder | 42.94 | -72.24 | 14 | 99 |
| LK1273 | New Hampshire | Hillsborough | HUH | Batchelder s.n. | 42.82 | -71.63 | 15 | 97 |
| LK1274 | Connecticut | New London | HUH | Anderson 930 | 41.53 | -72.11 | 15 | 43 |
| LK1275 | Florida | Levy | FLAS | Abbott 9118 | 29.07 | -82.72 | 15 | 113 |
| LK1276 | Kentucky | Madison | BEREA | Thompson 10809 | 37.58 | -84.3 | 14 | 8 |
| LK1277 | Tennessee | Blount | BEREA | Thompson 09535 | 35.01 | -83.02 | 15 | 9 |
| LK1278 | Missouri | Warren | FLAS | Abbott 26078 | 38.73 | -91.14 | 15 | 6 |
| LK1279 | Maine | Cumberland | FLAS | Abbott 25568 | 43.86 | -70.11 | 15 | 8 |
| LK1280 | Rhode Island | Newport | FLAS | Abbott 25493 | 41.58 | -71.21 | 15 | 8 |
| LK1281 | New Jersey | Sussex | FLAS | Abbott 25420 | 41.35 | -74.68 | 15 | 8 |
| LK1282 | Georgia | Whitfield | FLAS | Abbott 25360 | 34.83 | -85.04 | 15 | 8 |
| LK1283 | Florida | Suwannee | FLAS | Abbott 22568 | 30.04 | -83.01 | 15 | 11 |
| LK1293 | Connecticut | Middlesex | HUH | Richardson s.n. | 41.47 | -72.48 | 15 | 54 |
| LK1294 | Connecticut | Windham | HUH | Mehrhoff 19324 | 41.66 | -72.1 | 15 | 21 |
| LK1295 | Maine | Androscoggin | HUH | R.C.B.1 | 44.1 | -70.3 | 10 | 86 |
| LK1296 | Maine | Cumberland | HUH | Furbish | 43.78 | -70.31 | 10 | 3 |
| LK1297 | Maine | Franklin | HUH | Laferriere 3914 | 44.01 | -70 | 10 | 12 |
| LK1298 | Maine | Hancock | HUH | Hill 2443 | 44.27 | -68.57 | 15 | 103 |
| LK1299 | Maine | Lincoln | HUH | Brierly 1226a | 44.18 | -69.47 | 15 | 87 |
| LK1300 | Maine | Oxford | HUH | Wheeler 62/203 | 44.49 | -70.8 | 15 | 80 |
| LK1301 | Maine | York | HUH | True 223 | 43.41 | -70.75 | 11 | 84 |
| LK1302 | Rhode Island | Bristol | HUH | Countryman 22206 | 41.64 | -71.26 | 15 | 54 |
| LK1303 | Rhode Island | Washington | HUH | Bill s.n. | 41.43 | -71.58 | 15 | 91 |
| LK1304 | North Carolina | Cabrrus | NCU | Ahles 15914 | 35.43 | -80.63 | 11 | 62 |
| LK1305 | North Carolina | Camden | NCU | Ahles 44418 | 36.46 | -76.33 | 15 | 60 |
| LK1307 | North Carolina | Currituck | NCU | Ahles 44491 | 36.36 | -75.92 | 14 | 60 |
| LK1309 | North Carolina | Nash | NCU | Ahles 11730 | 36.02 | -78.12 | 15 | 62 |
| LK1310 | North Carolina | Randolph | NCU | Downs 13255 | 35.61 | -79.83 | 15 | 3 |
| LK1311 | South Carolina | Allendale | NCU | Ahles 10667 | 32.83 | -81.37 | 15 | 62 |
| LK1312 | South Carolina | Cherokee | NCU | Ahles 11283 | 35.12 | -81.77 | 14 | 62 |
| LK1313 | South Carolina | Clarendon | NCU | Radford 21114 | 33.84 | -80.03 | 12 | 61 |
| LK1314 | South Carolina | Horry | NCU | Bell 6166 | 33.69 | -79.01 | 15 | 61 |
| LK1315 | South Carolina | Lancaster | NCU | Ahles 27470 | 34.77 | -80.52 | 15 | 61 |
| LK1316 | South Carolina | Marlboro | NCU | Radford 9291 | 34.58 | -79.76 | 15 | 62 |
| LK1317 | Louisiana | Acadia | ULM | Allen 12961 | 30.47 | -92.44 | 15 | 34 |
| LK1318 | Louisiana | Assumption | ULM | Thomas 117546 | 29.87 | -91.1 | 15 | 28 |
| LK1319 | Louisiana | Avoyelles | ULM | Thomas 118893 | 30.86 | -92.15 | 15 | 28 |
| LK1320 | Louisiana | Beuregard | ULM | Bruce 119 | 30.5 | -93.31 | 15 | 33 |
| LK1321 | Louisiana | Bienville | ULM | Thomas 100242 | 32.42 | -92.88 | 14 | 31 |
| LK1322 | Louisiana | Caddo | ULM | Lewis 3475 | 32.52 | -93.75 | 12 | 36 |
| LK1323 | Louisiana | Caldwell | ULM | Marx 463 | 32.09 | -92.17 | 7 | 45 |
| LK1324 | Louisiana | Cameron | ULM | Thomas 90684 | 30.01 | -92.78 | 14 | 34 |
| LK1325 | Louisiana | Claiborne | ULM | Lewis 1328 | 32.95 | -92.97 | 15 | 40 |
| LK1326 | Louisiana | Grant | ULM | Thomas 150887 | 31.64 | -92.47 | 15 | 22 |
| LK1327 | Louisiana | Iberia | ULM | Thomas 132576 | 29.81 | -91.79 | 15 | 26 |
| LK1328 | Louisiana | Iberville | ULM | Lewis 1130 | 30.4 | -91.51 | 15 | 41 |
| LK1329 | Louisiana | Lafouche | ULM | Guidroz 164 | 29.78 | -90.82 | 14 | 45 |
| LK1330 | Louisiana | Livingston | ULM | Thomas 130440 | 30.46 | -90.99 | 15 | 26 |
| LK1334 | Ohio | Summit | US | Adreas 3422 | 41.08 | -81.64 | 12 | 39 |
| LK1335 | Virginia | Rapphannock | US | Walker 2254 | 38.79 | -78.16 | 15 | 80 |
| LK1336 | Virginia | Page | US | Fosberg 42383 | 38.53 | -78.44 | 15 | 56 |
| LK1337 | Virginia | Bath | US | Morton 1957 | 37.97 | -79.6 | 15 | 88 |
| LK1338 | Kentucky | Pendleton | US | Braun 4485 | 38.83 | -84.27 | 3 | 76 |
| LK1339 | Virginia | Albermarle | US | Fosberg 36419 | 38.23 | -78.72 | 15 | 63 |
| LK1340 | New York | Tompkins | US | Coville s.n. | 42.44 | -76.5 | 11 | 133 |
| LK1341 | Georgia | McDuffie | US | Bartlett 2593 | 33.48 | -82.52 | 12 | 107 |
| LK1342 | Ohio | Lorain | US | Ricksecker s.n. | 41.24 | -82.22 | 13 | 123 |
| LK1343 | New York | Onondaga | US | Brown 8022 | 43.05 | -75.97 | 15 | 84 |
| LK1354 | Ohio | Ashland | KSC | Culler s.n. | 40.92 | -82.16 | 15 | 113 |
| LK1355 | West Virginia | Pocahontas | KSC | Holland 9618 | 38.37 | -79.88 | 14 | 19 |
| LK1356 | Oklahoma | Ottowa | KSC | Grannerman s.n. | 39.99 | -94.65 | 15 | 44 |
| LK1372 | Georgia | McIntosh | USF | Smith 2268 | 31.51 | -81.37 | 2 | 109 |
| LK1373 | Indiana | Jay | USF | Franck 3966 | 40.5 | -84.94 | 15 | 3 |
| LK1374 | North Carolina | Carteret | USF | Scarboro s.n. | 34.72 | -76.67 | 15 | 53 |
| LK1375 | Ohio | Shelby | USF | Franck 556 | 40.35 | -84.42 | 11 | 11 |
| LK1376 | Oklahoma | McCurtain | USF | Stanford 3133 | 34.03 | -94.57 | 14 | 49 |
| LK1377 | South Carolina | Charleston | USF | Hill 29423 | 33.15 | -79.4 | 15 | 25 |
| LK1378 | Tennessee | Cocke | USF | Genelle 2748 | 36 | -83.1 | 13 | 41 |
| LK1379 | Virginia | Smyth | USF | Kral 11691 | 36.66 | -81.54 | 14 | 58 |
| LK1380 | Illinois | Ogle | USF | Sorenson 1206 | 41.94 | -89.23 | 15 | 6 |
| LK1381 | Illinois | Alexander | ILLS | Phillippe 25868 | 37.26 | -89.41 | 15 | 24 |
| LK1382 | Illinois | Bond | ILLS | Evers 39335 | 38.98 | -89.35 | 15 | 65 |
| LK1383 | Illinois | Boone | ILLS | Evers 47220 | 42.38 | -88.81 | 8 | 63 |
| LK1384 | Illinois | Bureau | ILLS | Evers 9273 | 41.36 | -89.49 | 13 | 70 |
| LK1385 | Illinois | Carroll | ILLS | Robertson 4423 | 42.06 | -90.1 | 15 | 31 |
| LK1386 | Illinois | Cass | ILLS | Phillippe 24060 | 40.02 | -90.11 | 15 | 24 |
| LK1387 | Illinois | Champaign | ILLS | Layden 16 | 40.11 | -88.34 | 15 | 41 |
| LK1388 | Illinois | Chrisitan | ILLS | Evers 107936 | 39.41 | -89.46 | 15 | 46 |
| LK1389 | Illinois | Clay | ILLS | Evers 112542 | 38.77 | -88.43 | 15 | 44 |
| LK1390 | Illinois | Cook | ILLS | Evers 34835 | 42.14 | -88.04 | 15 | 66 |
| LK1391 | Illinois | Dewitt | ILLS | Evers 64336 | 40.12 | -89.08 | 15 | 58 |
| LK1392 | Illinois | Edwards | ILLS | Edgin 4749 | 38.61 | -88.12 | 15 | 17 |
| LK1393 | Kentucky | Campbell | MU | Buddell 259 | 39.11 | -84.47 | 14 | 37 |
| LK1394 | Kentucky | Jackson | MU | Taylor 3540 | 37.54 | -84.19 | 13 | 35 |
| LK1395 | Kentucky | Mercer | MU | Taylor 15761 | 37.82 | -84.74 | 15 | 27 |
| LK1396 | Kentucky | Wolfe | MU | Vincent 13590 | 37.77 | -83.43 | 15 | 11 |
| LK1397 | Ohio | Cuyahoga | MU | Georgius 42 | 41.35 | -81.93 | 15 | 16 |
| LK1398 | Ohio | Franklin | MU | Schutte 03 | 40.07 | -83.01 | 15 | 15 |
| LK1399 | Ohio | Hamilton | MU | White 5591-1 | 39.23 | -84.51 | 14 | 27 |
| LK1400 | Ohio | Highland | MU | Sulgrove 90071334 | 39.1 | -83.43 | 14 | 28 |
| LK1401 | Ohio | Logan | MU | McCormac 3674 | 40.3 | -83.79 | 15 | 27 |
| LK1402 | Ohio | Mercer | MU | Trisel 111 | 40.68 | -84.64 | 13 | 28 |
| LK1403 | Ohio | Montgomery | MU | Dister s.n. | 39.77 | -84.19 | 14 | 17 |
| LK1404 | Ohio | Portage | MU | Stewart 193 | 41.23 | -81.38 | 15 | 33 |
| LK1405 | Ohio | Seneca | MU | Jones 69-5-23-367 | 41.24 | -82.93 | 14 | 49 |
| LK1406 | West Virginia | Boone | WVA | Craig s.n. | 38.07 | -81.82 | 15 | 113 |
| LK1407 | West Virginia | Preston | WVA | Grafton s.n. | 39.38 | -79.64 | 15 | 21 |
| LK1408 | West Virginia | Monongalia | WVA | Dawson s.n. | 39.67 | -79.96 | 14 | 39 |
| LK1409 | West Virginia | Barbour | WVA | Grafton s.n. | 39.19 | -79.89 | 15 | 17 |
| LK1410 | West Virginia | Ohio | WVA | Grafton s.n. | 40.15 | -80.71 | 15 | 15 |
| LK1411 | West Virginia | Wood | WVA | Grafton s.n. | 39.24 | -81.29 | 15 | 19 |
| LK1412 | West Virginia | Fayette | WVA | Grafton s.n. | 38.14 | -81.1 | 15 | 14 |
| LK1413 | West Virginia | Berkeley | WVA | Grafton s.n. | 39.51 | -78.17 | 15 | 14 |
| LK1414 | West Virginia | Upshur | WVA | Chapman 5 | 38.89 | -80.3 | 13 | 13 |
| LK1416 | West Virginia | Roane | WVA | Bartholomew 14 | 38.91 | -81.43 | 12 | 41 |
| LK1417 | Virginia | Mecklenburg | GMUF | Belden 1839 | 36.6 | -78.37 | 14 | 19 |
| LK1418 | Virginia | Lancaster | GMUF | Stanley and Miller s.n. | 37.64 | -76.37 | 15 | 45 |
| LK1419 | New York | Hamilton | PLAT | Keelan 111 | 43.67 | -74.7 | 15 | 23 |
| LK1420 | New York | Clinton | PLAT | Kretchman s.n. | 44.62 | -73.42 | 12 | 50 |
| LK1421 | Tennessee | Shelby | TENN | Browne 70M5.7 | 35.32 | -90.05 | 14 | 48 |
| LK1422 | Tennessee | Crockett | TENN | Heineke 2271 | 35.78 | -89.13 | 13 | 37 |
| LK1423 | Tennessee | Blount | TENN | Thomas s.n. | 35.74 | -83.72 | 15 | 54 |
| LK1424 | Tennessee | Obion | TENN | Guthrie 1487 | 36.43 | -89.32 | 14 | 32 |
| LK1425 | Tennessee | Dyer | TENN | Deneke 1672 | 36.13 | -89.43 | 15 | 39 |
| LK1426 | Tennessee | Carter | TENN | Pyne 93-272 | 36.41 | -82.24 | 12 | 25 |
| LK1427 | Tennessee | Johnson | TENN | Evans 43122 | 36.52 | -81.93 | 14 | 49 |
| LK1428 | Tennessee | Monroe | TENN | Maltev 53569 | 35.4 | -84.18 | 15 | 41 |
| LK1429 | Tennessee | Gibson | TENN | Chester 14650 | 36.3 | -88.69 | 15 | 14 |
| LK1430 | Tennessee | Marion | TENN | Blyveis 40 | 35.01 | -85.61 | 15 | 9 |
| LK1431 | Tennessee | Polk | TENN | Jacobs 83 | 35.05 | -84.54 | 13 | 35 |
| LK1432 | Tennessee | Carroll | TENN | Thompson 558 | 36.14 | -88.45 | 15 | 46 |
| LK1433 | West Virginia | Calhoun | MUHW | Dowell 109 | 38.86 | -81.12 | 12 | 26 |
| LK1434 | West Virginia | Kanawha | MUHW | Strickland 210 | 38.46 | -81.5 | 15 | 20 |
| LK1435 | West Virginia | Logan | MUHW | Bowen 18 | 37.8 | -81.8 | 15 | 18 |
| LK1436 | West Virginia | Raleigh | MUHW | Dobson 241 | 37.76 | -81.18 | 15 | 29 |
| LK1437 | West Virginia | Mercer | MUHW | Brumfield 199 | 37.5 | -81.12 | 2 | 35 |
| LK1438 | West Virginia | Mason | MUHW | West 134 | 38.72 | -81.97 | 0 | 37 |
| LK1442 | Oklahoma | Tulsa | EKY | Clark 23383 | 36.09 | -95.99 | 14 | 22 |
| LK1443 | Kentucky | Barren | EKY | Lapham 21 | 36.89 | -85.98 | 15 | 25 |
| LK1444 | Kentucky | Marion | EKY | Clark 23604 | 37.59 | -85.06 | 15 | 21 |
| LK1445 | Kentucky | McCreary | EKY | Shaw 391 | 36.63 | -84.53 | 15 | 19 |
| LK1446 | Kentucky | Trigg | EKY | Mowrer 4 | 36.68 | -88.05 | 11 | 23 |
| LK1447 | Kentucky | Henderson | EKY | Hannan 3606 | 37.72 | -87.76 | 14 | 38 |
| LK1448 | Kentucky | Edmonson | EKY | Elmore 781 | 37.17 | -86.32 | 15 | 49 |
| LK1449 | Kentucky | Whitley | EKY | Carter 67 | 36.81 | -84.19 | 15 | 24 |
| LK1450 | Kentucky | Laurel | EKY | Allen 94 | 37.05 | -84.02 | 15 | 19 |
| LK1451 | Kentucky | Union | EKY | Dreier s.n. | 37.63 | -87.87 | 15 | 42 |
| LK1452 | Virginia | Buckingham | VPI | Little 106155 | 37.49 | -78.55 | 12 | 67 |
| LK1453 | Virginia | Giles | VPI | Williams 10388 | 37.32 | -80.83 | 10 | 30 |
| LK1454 | Virginia | Pittsylavania | VPI | Massey 1188 | 36.74 | -79.47 | 15 | 81 |
| LK1455 | Virginia | Amelia | VPI | Lewis 813 | 37.32 | -77.97 | 13 | 81 |
| LK1456 | Virginia | Bland | VPI | Kral 12881 | 37.22 | -81.04 | 15 | 57 |
| LK1457 | Virginia | Floyd | VPI | Uttal 11893 | 36.99 | -80.36 | 15 | 42 |
| LK1458 | Virginia | Prince George | VPI | Bailey s.n. | 37.14 | -77.23 | 14 | 113 |
| LK1459 | Virginia | Amherst | VPI | Massey 2942 | 37.59 | -79.05 | 14 | 79 |
| LK1460 | Kentucky | Christian | APSC | Francis s.n. | 36.92 | -87.48 | 15 | 50 |
| LK1461 | Kentucky | Lyon | APSC | Ellis 1895 | 36.95 | -88.13 | 15 | 52 |
| LK1462 | Tennessee | Houston | APSC | Chester 2855 | 36.23 | -87.53 | 15 | 56 |
| LK1463 | Tennessee | Henry | APSC | Chester 9068 | 36.33 | -88.13 | 15 | 28 |
| LK1464 | Louisiana | Ouachita | APSC | Englandand 142 | 32.51 | -92.29 | 15 | 51 |
| LK1465 | Alabama | Choctaw | APSC | Harrell 114 | 31.84 | -88.16 | 13 | 11 |
| LK1466 | Tennessee | Maury | APSC | Estes 58 | 35.45 | -87.27 | 15 | 19 |
| LK1467 | South Carolina | Orangeburg | DUKE | Wilbur 73355 | 33.43 | -80.36 | 15 | 17 |
| LK1468 | South Carolina | Pickens | DUKE | Rodgers 531 | 35.05 | -82.7 | 0 | 76 |
| LK1469 | South Carolina | Spartanburg | DUKE | Etters 17 | 34.93 | -81.97 | 5 | 58 |
| LK1470 | Virginia | Rockbridge | DUKE | Wyatt 676 | 37.8 | -79.46 | 13 | 44 |
| LK1471 | Virginia | Southampton | DUKE | Wilbur 75931 | 36.81 | -76.96 | 15 | 16 |
| LK1472 | Virginia | Halifax | DUKE | Thomas 953 | 36.62 | -79.07 | 15 | 30 |
| LK1473 | Virginia | Washington | DUKE | Small s.n. | 36.64 | -81.61 | 9 | 126 |
| LK1474 | North Carolina | Alamance | DUKE | Baim 125 | 36.1 | -79.38 | 14 | 26 |
| LK1475 | North Carolina | Macon | DUKE | Mark s.n. | 35.18 | -83.56 | 14 | 62 |
| LK1476 | North Carolina | Hyde | DUKE | Wilbur 65855 | 35.6 | -76.23 | 15 | 22 |
| LK1477 | North Carolina | Harnett | DUKE | Wilbur 52070 | 35.47 | -78.93 | 15 | 29 |
| LK1478 | North Carolina | Richmond | DUKE | Wilbur 56064 | 35.05 | -79.52 | 12 | 28 |
| LK1479 | North Carolina | Rowan | DUKE | Batson 893 | 35.73 | -80.65 | 15 | 67 |
| LK1480 | North Carolina | Surry | DUKE | Correll 14656 | 36.51 | -80.59 | 12 | 70 |
| LK1481 | North Carolina | Wake | DUKE | Wilbur 52181 | 35.72 | -78.95 | 11 | 29 |
| LK1482 | North Carolina | Pamlico | DUKE | Wilbur 60880 | 35.14 | -76.96 | 15 | 26 |
| LK1483 | North Carolina | Mecklenburg | DUKE | Daggy 7656 | 35.47 | -80.94 | 13 | 67 |
| LK1484 | North Carolina | Person | DUKE | Wilbur 47667 | 36.44 | -78.83 | 15 | 30 |
| LK1485 | North Carolina | Pitt | DUKE | Wilbur 60187 | 35.68 | -77.49 | 15 | 26 |
| LK1486 | North Carolina | Gates | DUKE | Wilbur 62285 | 36.45 | -76.61 | 15 | 24 |
| LK1487 | North Carolina | Edgecombe | DUKE | Wilbur 67810 | 35.93 | -77.61 | 15 | 21 |
| LK1488 | North Carolina | Duplin | DUKE | Wilbur 55073 | 34.94 | -78.07 | 15 | 28 |
| LK1489 | North Carolina | Brunswick | DUKE | Wilbur 63974 | 34.27 | -78.11 | 15 | 23 |
| LK1490 | North Carolina | Bladen | DUKE | Wilbur 58427 | 34.82 | -78.56 | 15 | 27 |
| LK1491 | South Carolina | Dorchester | DUKE | Wilbur 75163 | 33.17 | -80.53 | 13 | 16 |
| LK1492 | South Carolina | Farifield | DUKE | Nelson 5361 | 34.29 | -81.08 | 15 | 31 |
| LK1495 | South Carolina | Georgetown | DUKE | Wilbur 77129 | 33.55 | -79.4 | 15 | 14 |
| LK1496 | South Carolina | Williamsburg | DUKE | Wilbur 77167 | 33.48 | -79.89 | 14 | 14 |
| LK1497 | Delaware | Sussex | DOV | Tucker s.n. | 38.62 | -75.65 | 15 | 34 |
| LK1498 | Delaware | New Castle | DOV | Baereurodt s.n. | 39.69 | -75.75 | 9 | 81 |
| LK1499 | Delaware | Kent | DOV | McAvoy 4841 | 38.99 | -75.48 | 15 | 18 |
| LK1500 | Rhode Island | Newport | APSC | Laferriere 3691 | 41.54 | -71.27 | 15 | 15 |
